# Perinatal Photoperiod Has Long-Term Effects on the Rest-Activity Cycle and Sleep in Male and Female Mice

**DOI:** 10.1177/07487304241302547

**Published:** 2024-12-18

**Authors:** Rick van Dorp, Tom Deboer

**Affiliations:** Laboratory of Neurophysiology, Department of Cell and Chemical Biology, Leiden University Medical Center, Leiden, The Netherlands

**Keywords:** perinatal photoperiod, C3H mice, activity profile, activity duration, sleep EEG, sex differences

## Abstract

Environmental light conditions during development can have long-lasting effects on the physiology and behavior of an animal. Photoperiod, a clear example of environmental light conditions, is detected by and coded in the suprachiasmatic nucleus. It is therefore possible that differences observed in behavior in adulthood after exposure to different perinatal photoperiods are caused by lasting changes in the suprachiasmatic nucleus or alternatively, in other nuclei affected by perinatal photoperiod. It can then be expected that behavior with strong circadian aspects, like rest-activity and sleep, are affected by difference in photoperiod during development as well. To investigate this further, we exposed mice to different photoperiods during their development in the womb until weaning (long: 16 h of light, 8 h of darkness; short: 8 h of light, 16 h of darkness). After weaning, the animals were exposed to a 12 h:12 h light:dark cycle for at least 3 more weeks and some animals were subsequently exposed to constant darkness. We assessed their rest-activity patterns by recording voluntary locomotor activity and used EEG recordings to determine sleep architecture and electroencephalographic spectral density. Perinatal long photoperiod animals showed a shorter duration of locomotor activity than short photoperiod-developed mice in a 12:12 light-dark cycle. This difference disappeared in constant darkness. In the light phase, that is, during the day, perinatal long photoperiod mice spent less time awake and more time in NREM sleep than short photoperiod-developed mice. No effects of perinatal photoperiod were observed in the EEG spectral density or in response to sleep deprivation. We see lasting differences in behavioral locomotor activity and sleep in female and male mice after exposure to different perinatal photoperiods. We conclude that perinatal photoperiod programs a developing mammal for different external conditions and changes brain physiology, which in turn results in long-lasting, possibly even permanent, changes in the sleep and locomotor activity.

A large part of the world is exposed to seasonal variation of daylength and many animals respond to this with different types of seasonal adaptations in behavior and physiology. During the developmental period, daylength can have long-lasting effects. For instance, exposure to constant light during development in mice affects the immune system (Mizutani et al., 2017) and affects adult hormonal homeostasis (Brooks et al., 2011; Coleman and Canal, 2017). Constant light conditions are rather extreme and only occur naturally in the polar regions, but there is also solid evidence for developmental effects of moderate differences in daylength (photoperiod). Before birth, in utero, an important zeitgeber is melatonin (Goldman, 2003; Weaver and Reppert, 1986), which is produced and secreted during darkness. The melatonin profile strongly responds to photoperiod (Bartness and Goldman, 1989). This response is also detected in utero, and it has been shown that changes in the maternal melatonin profile can affect clock gene expression in the offspring (Seron-Ferre et al., 2007; Torres-Farfan et al., 2006). Moreover, it has been established that the maternal melatonin profile plays a role in physiological differences between offspring born in different photoperiods (van Dalum et al., 2019), to prepare them for future changes in the environment related to changing seasons. This maternal photoperiodic programming induces faster growth and maturation of voles born early in the season, allowing them to reproduce within the same mating season as they were born in (Horton, 1984; Lee, 1993) and it plays a role in establishing the appropriate seasonal physiology in mammals with longer developmental periods, like sheep (Ebling et al., 1989) and red deer (Adam et al., 1994) who mate in autumn and give birth in spring.

At birth, a significant proportion of the circadian system is not fully developed (reviewed for the SCN in: Astiz and Oster, 2018; Landgraf et al., 2014) and the maturation of the system continues postnatally. Exemplary to this continued postnatal development in humans is the increase in daily rhythmic strength in rest-activity behavior in infants (Jenni et al., 2006). After birth, the offspring is no longer supplied with maternal melatonin through the placenta, and although melatonin might still be present in milk (Hausler et al., 2024; Rowe and Kennaway, 2002), the role of postnatal melatonin in maternal photoperiodic programming is suggested to be negligible in rodents (Horton, 1985; Rowe and Kennaway, 2002; van Dalum et al., 2019). Instead, the offspring is exposed to the external light-dark cycle and is presumably influenced directly by daylength.

The long-term effects of photoperiod during the perinatal period have been studied previously. In humans, season of birth has been associated with differences in prevalence of several neuronal and psychological disorders (Foster and Roenneberg, 2008), like schizophrenia, autism, and depression. It was also shown that photoperiod during development is related to chronotype in adulthood, with later chronotype being associated with birth during increasing daylength (Vollmer et al., 2012). Other studies have found associations between perinatal photoperiod (PNP) and the incidence of depression in adulthood (Devore et al., 2018; Lewis et al., 2024). Possible underlying changes in physiology have been studied in mice, where development in different photoperiods leads to differences in affective-like behavior (Green et al., 2015). Mice developed in a long photoperiod showed a higher spontaneous and evoked spike rate and a higher serotonin and noradrenalin concentration in the midbrain. In addition, exposure to a short photoperiod during development led to a longer behavioral free-running rhythm, a longer in vitro *Per1::GFP* expression peak in the SCN and a longer endogenous rhythm of *Per1::GFP* expression in individual neurons (Ciarleglio et al., 2011).

Photoperiodic information is detected by, coded, and stored in the SCN (VanderLeest et al., 2007, 2009). It is therefore possible that differences observed in behavior in adulthood after exposure to different PNPs, are caused by lasting changes in the SCN or alternatively, affects other nuclei downstream of the SCN. Therefore, behavior with strong circadian aspects, like rest-activity and sleep, may be affected by difference in photoperiod during development as well.

To investigate whether PNP indeed affects circadian behavior, we set out to quantify the long-term effects of PNP on the behavioral rest-activity pattern, sleep architecture, and the sleep electroencephalogram (EEG) in mice. We exposed male and female mice to a short (SP; 8 h of light, 16 h of darkness), a long (LP; 16 h of light, 8 h of darkness), or an intermediate photoperiod (12 h of light, 12 h of darkness) during their development and subsequently assessed their rest-activity patterns in voluntary wheel-running activity after at least 3 weeks in 12 h:12 h light-dark (LD) conditions and in constant darkness (DD). We used EEG to determine sleep architecture during baseline, a 6-h sleep deprivation, and an 18-h recovery time in LD.

## Methods

### Animals

**Figure 1.**
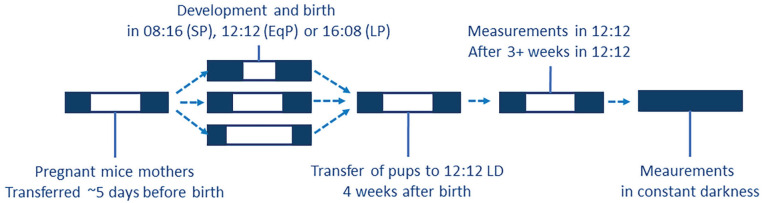
Scheme of the exposure to perinatal photoperiods. From visible pregnancy, the mothers were housed in a short photoperiod (8 h of light, 16 h of darkness), long photoperiod (16 h of light, 8 h of darkness), or an equal/neutral photoperiod (12 h of light and darkness) until the pups were weaned at 4 weeks of age. After weaning, the pups remained group-housed with same-sex pups from the same nest in a 12 h:12 h light:dark schedule for at least 3 weeks. The first measurements took place after at least 3 weeks of 12:12. Animals that were measured in constant darkness (DD) were exposed to DD after the measurements in 12:12 and were in DD for at least 2 weeks before measurement.

All animal experiments were approved by the Central Committee of Animal Research (the Netherlands) and were carried out in accordance with the EU directive 2010/63/EU on the protection of animals used for scientific purposes. Sixty-four female and 60 male C3H/HeNHsd were bred in the LUMC animal facility. At visible pregnancy (average of 5 days before birth per nest), the mothers were housed in a short photoperiod (8 h of light, 16 h of darkness; 28 female, 33 male pups born) or long photoperiod (16 h of light, 8 h of darkness; 36 female, 27 male pups born) until the pups were weaned at 4 weeks of age (Figure 1). After weaning, the pups remained group-housed with same-sex pups from the same nest in a 12 h:12 h light:dark schedule for at least 3 weeks. The first measurements took place after at least 3 weeks of 12:12. Animals that were measured in constant darkness (DD) were exposed to DD after the measurements in 12:12 and were in DD for at least 10 days. For the EEG analysis, 27 animals (SP: 7 females, 5 males; LP: 8 females, 7 males) were bred in the same conditions and an additional group of 12 (5 females) was raised in 12:12 (EqP). All breeding animals were obtained from Envigo (Envigo Research Models and Services; Horst, the Netherlands), and all animals were housed in Plexiglas cages with food and water available ad libitum and a light intensity of 50-100 lux at bottom of cage, in a temperature-controlled (21-22 °C) and humidity-controlled (35%-65%) environment.

### Rest-Activity Recording

The mice were solitary housed with a running wheel to record voluntary wheel-running behavior for at least 10 days in 12:12 and subsequently at least 10 days in DD to record free-running behavior and period. All locomotor behavior was recorded and converted to activity profiles with Clocklab (v6.1.05, Actimetrics, Illinois, USA). Activity profiles in DD were aligned at circadian time 12 (CT12) and activity onset was defined as the 60 min with the biggest difference in activity to the next 60 min. For animals that seemed to not align right, the beginning of the first 120 min above the average activity was used as onset. Peak of activity duration was defined as the consecutive time above 50% of individual maximum values, allowing on average 22 min of activity below 50% within the active time window.

### EEG/EMG Electrode Implantation Surgery

EEG and electromyogram (EMG) implantation procedures were as previously described (Panagiotou et al., 2017; van Dorp et al., 2024). In short, the mice were anesthetized with a mix of ketamine (100 mg/kg), xylazine (10 mg/kg), and atropinesulfate (0.1 mg/kg) and fixed in a stereotact. Two holes were drilled (right hemisphere, 2 mm lateral to midline, 2 mm posterior to bregma; cerebellum, at midline, 2 mm caudal to lambda) for EEG electrodes and 2 holes were drilled for stabilizing screws. Two EMG electrodes were inserted between the skin and neck muscle. The 2 EEG and 2 EMG wires were inserted in a pedestal (Plastics One, Roanoke, Virginia, USA), which was fixed to the skull with dental cement. At the end of the surgery, a cap was screwed on the pedestal to seal the connector holes and prevent cage litter from entering the connector holes. After surgery, the mice recovered in solitary housing for at least 7 days before entering the recording setup.

### EEG/EMG Recording and Sleep Deprivation

In the recording cages, animals were connected to a cable, which was connected to a counterbalanced swivel system to allow for free movement within the recording cage. Light, humidity, and temperature in the recording cage were comparable to the home cage and food and water were available ad libitum. The signal was amplified ~2000 times and was filtered through an ACQ-7700 system (Data Sciences International, New Brighton, MN, USA) with a low pass filter of 100 Hz and subsequently recorded on a local computer with Ponemah v5.53 (DSI), with a primary sampling rate of 250 Hz and a secondary sampling rate of 125 Hz. Files were then prepared for scoring by filtering the 50.0 Hz powerline and by filtering EEG channels with a band pass of 0.5-25.0 Hz and filtering EMG with a band pass of 3.0-25.0 Hz. The recordings were done in 12:12 and started at lights-on, first for 24 h to establish a baseline, immediately followed by another 24 h with a 6-h sleep deprivation starting at lights-on. To investigate possible differences in sleep homeostatic responses (Borbély et al., 2016), animals were sleep deprived by gentle handling as previously described (Panagiotou et al., 2017; van Dorp et al., 2024). When the animals appeared to fall asleep or when the EEG exhibited slow waves, the animals were woken up by noise, or introducing new bedding, food, water, or cage enrichment.

### Analysis

Activity profiles were compiled for 1-min bins in Clocklab data collection software (Actimetrics, Wilmette, IL, USA) and averaged in 1-h bins. Rhythmic strength was determined during DD by taking the difference in a F-periodogram from the level of significance (Jenni et al., 2006; Panagiotou and Deboer, 2020; Stenvers et al., 2016). Intradaily variability was determined during LD by Clocklab. Phase-shifting capability was approximated by applying a 15-min light pulse at CT14 after at least 2 weeks in DD.

EEG was scored manually in epochs of 4 sec into 3 different states: waking, rapid eye movement (REM) sleep, and non-REM (NREM) sleep. Epochs that contained artifacts were excluded from analysis of power spectra, but vigilance states could almost always be determined (max 0.18% artifacts per animal). Scored data were analyzed as percentage of time spent in a state for 1-h averages. Episode duration was determined by dividing the total time in a state by the amount of episodes. Spectral analysis was performed using a fast Fourier transform with 0.5-Hz bins from 0.5 to 5.0 Hz and in 1.0-Hz bins from 5.0 to 25.0 Hz. Afterward, brain activity was further analyzed per hour for slow wave activity (SWA) relative to the baseline average per animal.

In SPSS (v29, IBM corp) or GraphPad prism (v9.3.1 GraphPad Software, LLC), an analysis of variance (ANOVA; or a generalized linear model when a dataset had missing values) was performed with the factors *PNP, sex*, and, when present, *time* (zeitgeber time, ZT or CT, with hour 12 as activity onset for both) and the interaction of 2 or 3 factors for the analysis of rest-activity, vigilance state distribution, episode duration and distribution, and EEG power density spectra. When *time* was a factor, different time points were considered as repeated measures. Another general linear model (GLM) was performed with the additional factor *day* for the analysis of the effect of sleep deprivation (excluding the data obtained during sleep deprivation and the corresponding baseline period). Post hoc paired or unpaired *t* tests were performed where appropriate if the GLM showed significance for the factor *PNP* or if an interaction with *time* was significant. Graphs were produced using GraphPad prism.

## Results

### Activity Profile and Circadian Behavior Parameters

**Figure 2.**
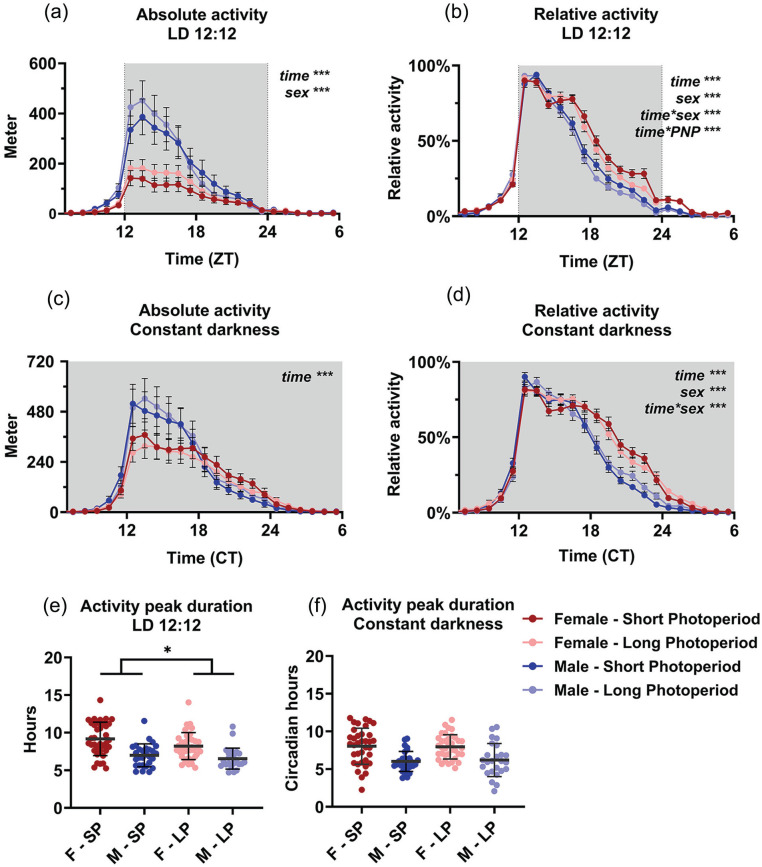
Perinatal photoperiod affects wheel-running activity patterns during adulthood. Wheel-running activity of female (red) and male (blue) mice developed in different photoperiods (SP: 08:16, Dark colors; LP: 16:08, light colors). Activity profiles (average hourly values of 10-12 days) with absolute activity expressed in distance ran in meters (a and c) and relative to the maximum per individual animal (b and d). The active phase is centered for visibility, and constant darkness values are aligned at CT12 (see Methods). A 3-way ANOVA with repeated measures indicated that male mice ran more during LD, but not during DD and that SP animals had a different pattern of activity in female and male mice in LD. Female and male mice from long perinatal photoperiods showed a shorter peak duration of activity in LD (*p* = 0.030), but not in DD (e and f). Mean with SEM in panels a, b, c, and d; mean with SD in panels e and f. *p < 0.05, ***p < 0.001.

Both absolute (wheel counts/hour) and relative (percentage of the maximum per animal) values of wheel-running activity were analyzed to emphasize the absolute running distance and the distribution of running activity separately. In absolute running distance, significant effects of time in LD and DD (*p* < 0.0001) and sex in LD (*p* < 0.0001) were observed (Figure 2a and 2c). Female mice showed significantly less activity in LD (SP females: 1.08 km/day; LP females: 1.41 km/day; SP males: 2.57 km/day; LP males: 2.67 km/day; *p* = 0.0002), but this difference between the sexes disappeared in DD (*p* = 0.2663). No effect of PNP was observed with regard to absolute activity.

In the normalized activity data (Figure 2b and 2d), a 3-way ANOVA for sex, time, and PNP indicated a significant effect of time (*p* < 0.0001), sex (*p* < 0.0001), and the interaction of time and sex (*p* < 0.0001) in both LD and DD. PNP itself did not show a significant effect (LD: *p* = 0.0757; DD: *p* = 0.4423), but the interaction of time and PNP was significant in LD (*time × PNP: p* < 0.0001; DD *p* = 0.499), indicating that the animals had a different distribution of behavior over the day depending on PNP. Post hoc *t* tests between the LP and SP group indicated that the largest difference in relative activity was between ZT 19 and 23 (*p* < 0.047) in LD. The 3-way interaction of time, sex, and PNP was significant in DD (*p* = 0.0214; LD *p* = 0.8023), suggesting a different effect of PNP between males and females on activity pattern in DD. Further analysis of activity duration showed that LP animals had a shorter peak of locomotor activity (7.55 ± 1.83 h) than SP animals (8.22 ± 2.22 h; *p* = 0.030, Figure 2e) in LD, but not in DD (*p* = 0.892; Figure 2f), and that females had a longer peak of activity (8.67 ± 2.04 h) than males (6.77 ± 1.46 h; *p* < 0.001; Figure 2e).

**Figure 3.**
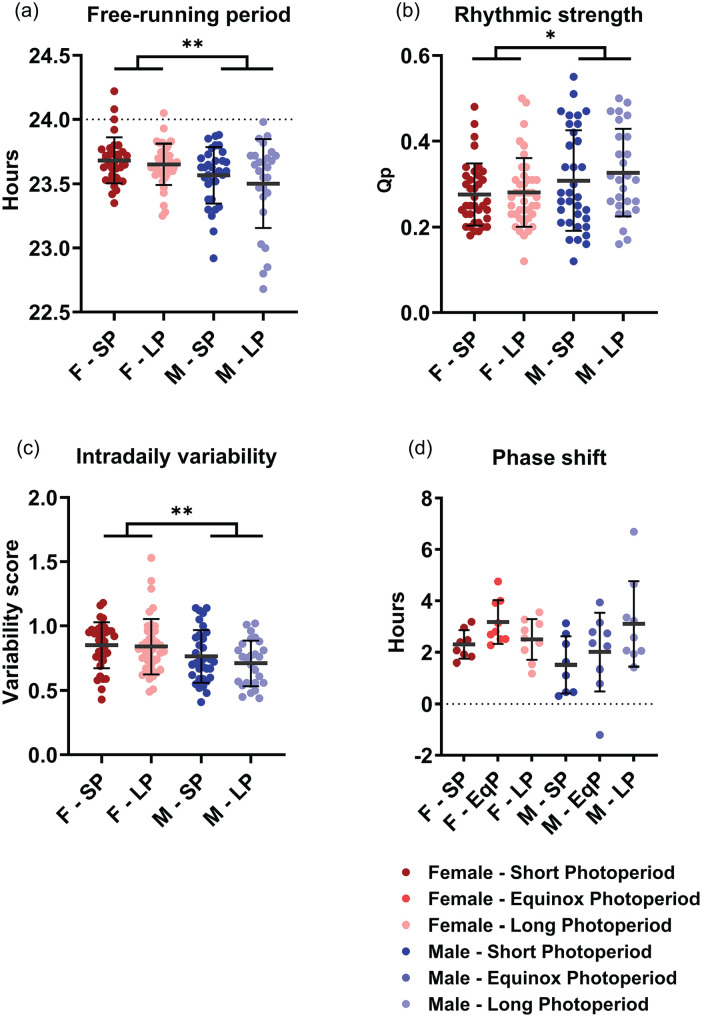
Perinatal photoperiod does not affect circadian parameters. Four circadian parameters measured in DD in female (red) and male (blue) mice developed in different photoperiods (SP: 08:16, Dark colors; LP: 16:08, light colors). A 2-way ANOVA indicated that female mice have a longer free-running rhythm (a), lower rhythmic strength (b), and higher intradaily variability (c). No difference in phase-shifting capacity was found (d). Perinatal photoperiod does not cause significant differences in any of the variables, but the variation of free-running period is larger in LP-developed males than in SP-developed males (*p* = 0.032). Mean with SD. *p < 0.05, **p < 0.01.

From the activity data in DD, we also obtained the free-running period, rhythmic strength, intradaily variability and, in a smaller separate group, phase-shifting capacity (Figure 3). None of these resulted in significant effects of PNP, except that male mice showed a larger variance in free-running period in the LP condition compared to the SP condition (*p* = 0.032, Figure 3a). A 2-way ANOVA indicated that the free-running period in DD was significantly longer in the female mice than in the male mice (females: 23.68 h ± 0.16 h; males: 23.55 h ± 0.27 h; *p* = 0.001; Figure 3a). Sex was not a significant factor in the phase-shifting capacity to light (sex: *p* = 0.172; PNP: *p* = 0.078; Figure 2d), but females did show a higher intradaily variability score (0.843 ± 0.202) than males (0.737 ± 0.184; *p* = 0.002) and a lower rhythmic strength (0.281 ± 0.078) than males (0.314 ± 0.109; *p* = 0.036).

### Sleep and Waking

**Figure 4.**
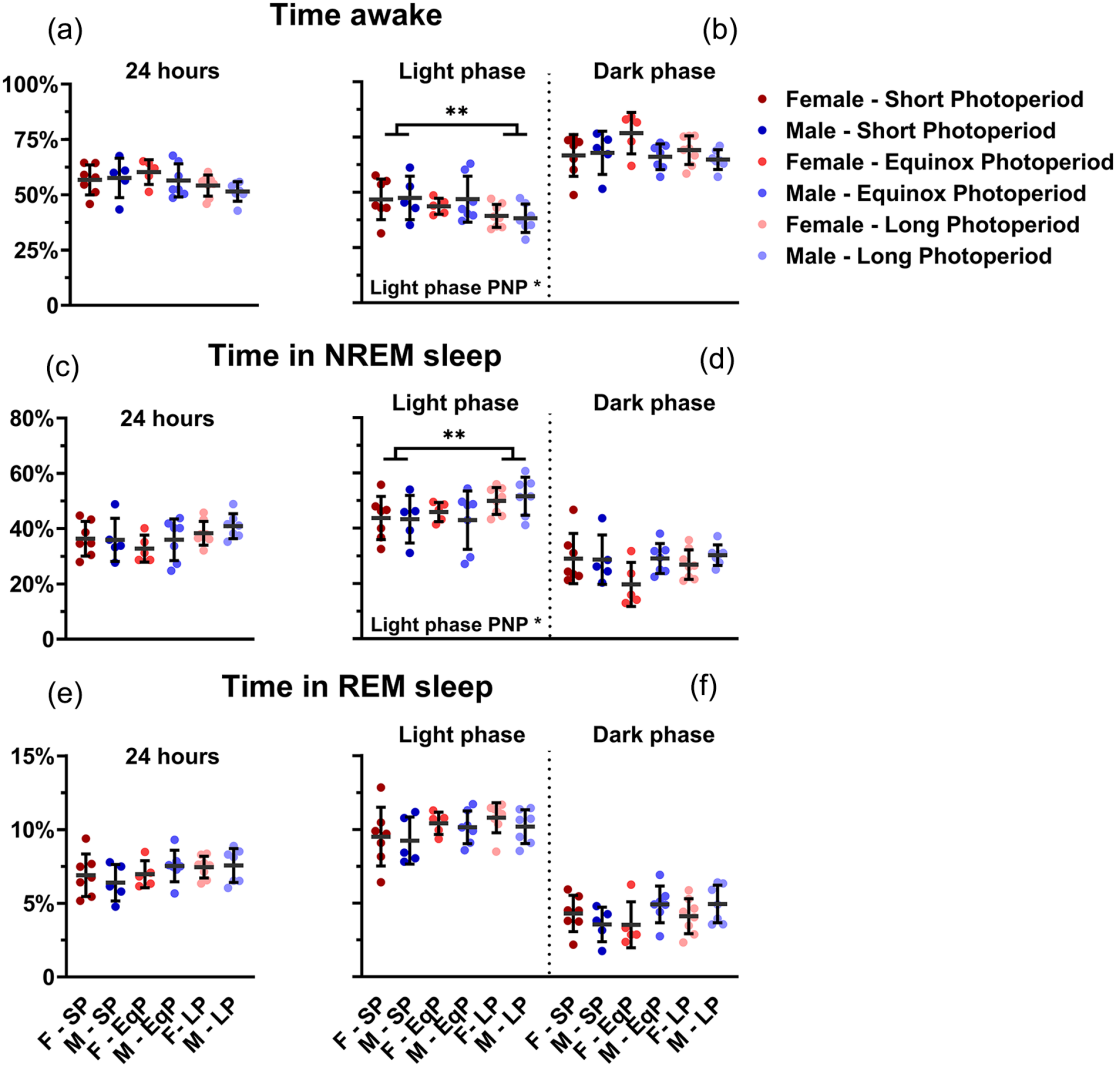
Mice developed in a long photoperiod spent more time asleep in the light phase. Vigilance state distribution in female (red) and male (blue) mice developed in different photoperiods (SP: 08:16, Dark colors; LP: 16:08, light colors). 24-h averages of percentage of time spent awake (a), in NREM sleep (c), and in REM sleep (e) and 12-h average of the light and dark phase in the same order (b, d, f). A 2-way ANOVA indicated that the LP-developed animals of both sexes spent less time awake (*p* = 0.023) and more in NREM sleep (*p* = 0.032) in the inactive (light) phase and post hoc *t* tests for the light phase showed that LP-developed mice spent less time awake (*p* = 0.007) and more time in NREM sleep (*p* = 0.011) than SP-developed mice. No difference was observed in the 24-h values or in the dark phase. Mean with SD. **p < 0.01.

**Figure 5.**
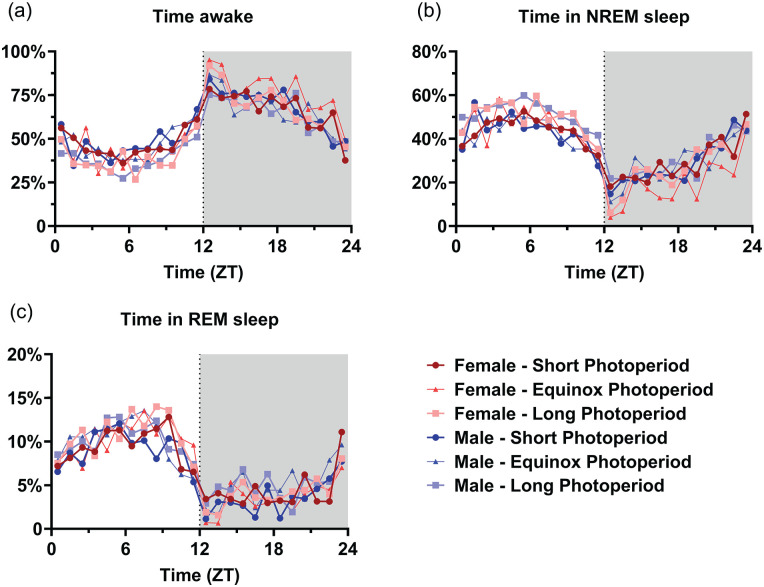
Mice developed in a long photoperiod did not spent significantly more time asleep per hour. Time course of 1-h vigilance state values in female (red) and male (blue) mice developed in different photoperiods (SP: 08:16, dark-colored squares; EqP: 12:12, intermediate brightness triangles; LP: 16:08, light-colored circles). Averages of percentage of time spent awake (a), in NREM sleep (b), and in REM sleep (c). A 3-way ANOVA corrected for repeated measures indicated no significant difference between the perinatal photoperiod groups or females and males, but time was a significant factor in all groups. For easier visibility, only the mean values are plotted.

No difference between PNP was observed in the 24-h total time spent awake (SP: 57.1% ± 7.3%; EqP: 58.1% ± 6.8%, LP: 53.0% ± 4.7%, *p* = 0.074; Figure 4a), in NREM sleep (SP: 36.2% ± 6.6%, EqP: 34.6% ± 6.5%, LP: 39.5% ± 4.4%, *p* = 0.082; Figure 4c) or in REM sleep (SP: 6.7% ± 1.3%, EqP: 7.3% ± 1.0%, LP: 7.5% ± 0.9%, *p* = 0.151; Figure 4e). In the 12-h data representing the light and dark phase, PNP was a significant factor in the amount of waking and NREM sleep in the light phase (waking: *p* = 0.023; NREM sleep: *p* = 0.032; Figure 4b, 4d, 4f), but not in the amount of REM sleep (*p* = 0.102) or in the dark phase (waking: *p* = 0.275; NREM sleep: *p* = 0.211; REM sleep: *p* = 0.485; Figure 4b, 4d, and 4f). Post hoc *t* tests for the light phase showed that LP-developed mice spent less time awake (SP: 47.1% ± 9.0%; LP: 38.8% ± 5.7%; *p* = 0.007) and more time in NREM sleep (SP: 43.5% ± 7.8%; LP: 50.7% ± 5.7%; *p* = 0.011) than SP-developed mice. No significant difference was observed between female and male mice in either phase (Light phase: wake *p* = 0.720; NREM sleep *p* = 0.820; REM sleep *p* = 0.387; Dark phase: wake *p* = 0.070; NREM sleep *p* = 0.071; REM sleep *p* = 0.246). When investigating 1-h values of vigilance state distribution, there appeared to be no effect of PNP on the amount of time spent awake (*p* = 0.085; Figure 5a), in NREM sleep (*p* = 0.128; Figure 5b), or REM sleep (*p* = 0.069; Figure 5c). This indicates that the effect of PNP on vigilance state distribution is spread through the entire light phase and cannot be specifically ascribed to a certain hour or period of the light phase.

Despite differences in the amount of NREM sleep and waking in the light period, there was no significant effect of PNP on amount of episodes or time spent in an episode. However, there was also no significant effect of sex or photoperiod in episode amount and duration (Supplementary Fig. 1) in the light phase. In the dark phase, male animals had more waking episodes (274.1 ± 58.81; *p* = 0.019), NREM sleep episodes (278.0 ± 54.99; *p* = 0.013), and REM sleep episodes (79.37 ± 31.94; *p* = 0.005) than females (waking episodes: 228.3 ± 65.88; NREM sleep episodes: 231.5 ± 63.99; REM episodes: 51.80 ± 19.65). Consequently, male animals had shorter waking (1.81 ± 0.40 min; *p* = 0.005) and REM sleep episodes (4.41 ± 0.95 min; *p* = 0.003) than females (waking: 2.42 ± 0.88 min; REM sleep: 5.93 ± 1.71 min), but NREM sleep episode length was not significantly different between the sexes (females: 9.05 ± 2.47 min; males: 8.53 ± 2.58 min; *p* = 0.585).

### Electroencephalogram

**Figure 6.**
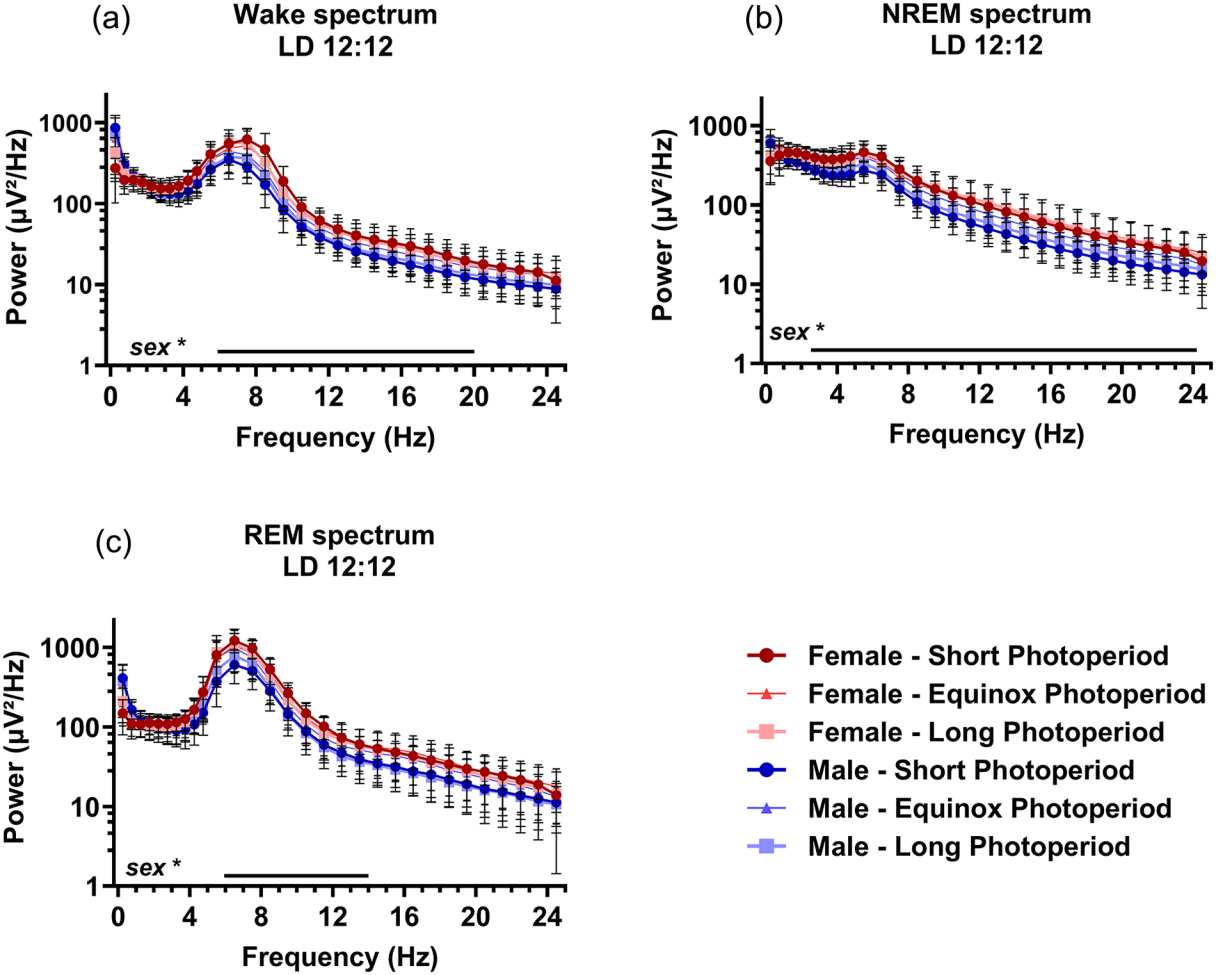
Sex, but not perinatal photoperiod, affects the EEG spectral power density. EEG power density spectra in female (red) and male (blue) mice developed in different photoperiods (SP: 08:16, dark-colored squares; EqP: 12:12 intermediate brightness triangles; LP: 16:08, light-colored circles). Power density during waking (a), NREM sleep (b), and REM sleep (c) in 0.5 Hz bins from 0.5 to 5.0 Hz and in 1 Hz bins from 5.0 to 25.0 Hz. An ANOVA accounting for repeated measures indicated that females had a slightly higher power density during waking between 6.0 and 20 Hz, NREM sleep between 2.5 and 24 Hz, and REM sleep between 6.0 and 14 Hz, range of significant sex differences indicated by a line below. Mean with SD. *p < 0.05.

EEG power density spectra were calculated and compared between males and females and between animals raised under different photoperiods. The spectra of all vigilance states showed a prominent peak in the theta range, as previously found in this mouse strain (van Dorp et al., 2024). No effect of PNP on spectral density was observed. Compared to males, female mice showed a significantly higher spectral power density in waking between 6.0 and 20 Hz (*p* < 0.048; Figure 6a), NREM sleep between 2.5 and 24 Hz (*p* < 0.045; Figure 6b), and REM sleep between 6.0 and 14 Hz (*p* < 0.047; Figure 6c).

**Figure 7.**
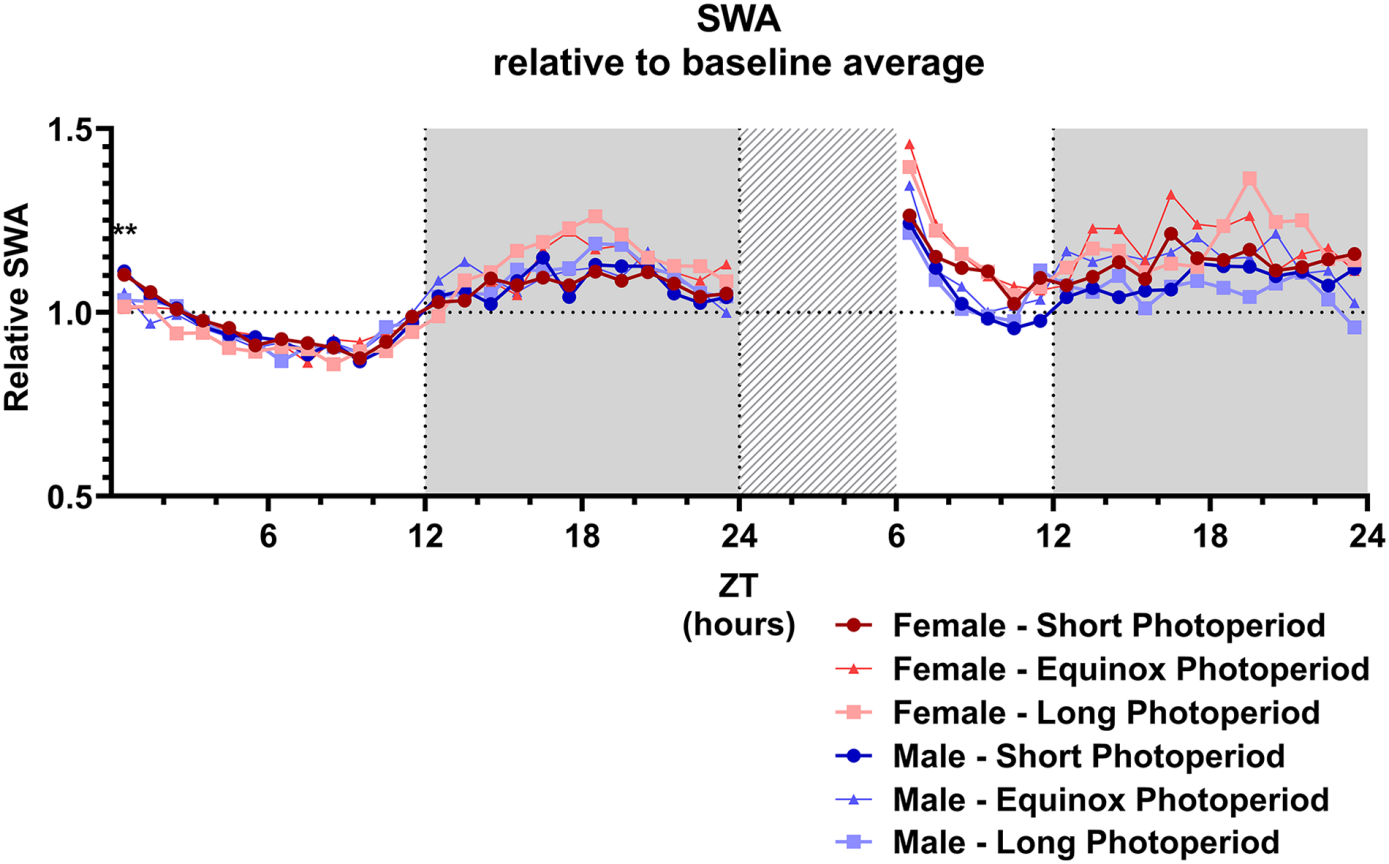
Slow wave activity is not affected by sex or perinatal photoperiod. Slow wave activity (0.5-4.0 Hz; SWA) during NREM sleep in female (red) and male (blue) mice developed in different photoperiods (SP: 08:16, dark-colored squares; EqP: 12:12 intermediate brightness triangles; LP: 16:08, light-colored circles) during the baseline day and after a 6-h sleep deprivation. A GLM with post hoc *t* tests indicated that, during the baseline, only at ZT1 the animals developed in a short photoperiod showed an increased relative SWA, but there was no difference between the photoperiods at any other point during the baseline or after the sleep deprivation. For easier visibility, only the mean values are plotted. **p < 0.01.

We subsequently analyzed EEG SWA in NREM sleep as a marker for sleep intensity (Borbély et al., 2016). Only at ZT 1, a difference during baseline in relative SWA was indicated by a general linear model (GLM) comparing the PNP groups (SP: 1.11 ± 0.09; EqP: 1.04 ± 0.07; LP: 1.02 ± 0.07; *p* = 0.005; Figure 7), but there was no difference between the PNP groups at other time points of the baseline day. Female and male mice showed similar levels of relative SWA throughout the entire baseline day.

### Effect of Sleep Deprivation

**Figure 8.**
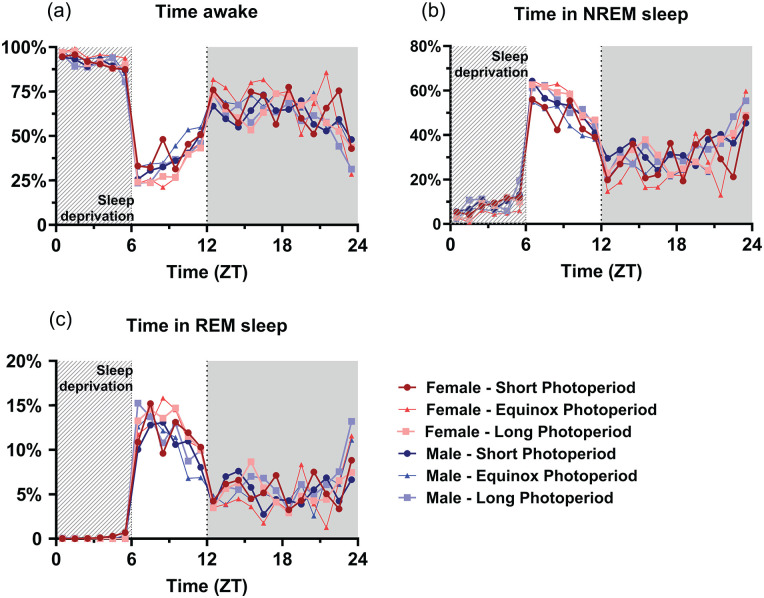
Perinatal photoperiod does not affect vigilance state distribution in response to a 6-h sleep deprivation. Time course of 1-h vigilance state values in response to a 6-h sleep deprivation in female (red) and male (blue) mice developed in different photoperiods (SP: 08:16, dark-colored squares; EqP: 12:12, intermediate brightness triangles; LP: 16:08, light-colored circles). Averages of percentage of time spent awake (a), in NREM sleep (b), and in REM sleep (c). A GLM with post hoc *t* tests indicated that the sleep deprivation increased NREM and REM sleep and decreased waking (*p* < 0.0028), but no interaction was found with sex or PNP. For easier visibility, only the mean values are plotted.

A general linear model to investigate the effect of sleep deprivation indicated it a significant factor for time spent awake, in NREM sleep, or REM sleep (*p* < 0.001 for all vigilance states; Figure 8). This means that the mice had a different vigilance state distribution after the sleep deprivation compared to the same time period during the baseline day, but no interaction was found with sex or PNP. Post hoc *t* tests indicated the most significant difference between the baseline and sleep deprivation condition from ZT 7 to 14 for waking and NREM sleep and from ZT 13 to 15 for REM sleep (*p* < 0.0028 for all mentioned time points). As expected, the sleep deprivation also increased relative SWA in NREM sleep in all groups between ZT 6 and 10 (*p* < 0.001; Figure 7), but there was no significant difference between the PNP groups or males and females in SWA after a sleep deprivation.

## Conclusion

We exposed mice to different photoperiods during perinatal development and subsequently housed them in a 12 h:12 h light-dark cycle for at least 3 more weeks. We found long-term effects of PNP on free-running voluntary locomotor activity rhythms, on the time course of rest-activity over the day, and on sleep-wake distribution.

We observed differences in the duration of running wheel activity in animals from different PNPs, and LP-developed animals had a shorter duration of peak activity than SP-developed animals. The findings were similar in males and females. The observed difference is similar to the difference change found in animals exposed to a long or short photoperiod at the time of measurement (as in Leclercq et al., 2021) and is reminiscent to after-effects in constant darkness, initially described by Pittendrigh and Daan (1976). Importantly, after exposure to the PNP, the mice in our experiment were exposed to at least 3 weeks of 12:12 LD. Exposing the mice to 12:12 gives the mice the opportunity to adapt to this light regime. Because the effects are still visible after adaptation to 12:12, this suggests that the effects we observe in duration of activity are both long-lasting and stable enough to persist after exposure to a different light regime. This indicates the existence of a sensitive period during development regarding photoperiodic adaptation. Although not specifically addressed in our study, the change in duration of activity may suggest involvement of changes in neuronal phase relations in the SCN, previously shown to reflect photoperiodic changes in activity profiles (Ciarleglio et al., 2011; VanderLeest et al., 2007). Alternatively or simultaneously, the observed effects might stem from changes to other brain nuclei that are affected by photoperiod during development like the dorsal raphe nuclei (Green et al., 2015) or the nucleus accumbens (Jameson et al., 2023). Interestingly, in constant darkness, the effect of PNP on activity peak width disappeared. This might indicate different responses to the light-dark cycle between the PNP groups, but it may also result from the inherently larger variability in activity data obtained from animals in constant darkness. In addition, the decreased effect might also stem from the variation of duration of exposure to 12:12 LD before measurement. The exposure to DD came after the 12:12 LD condition, and longer exposure to 12:12 could possibly equalize the effects in the different photoperiod groups by increasing the factor of “time since exposure to perinatal photoperiod.” Further research could investigate the effect of duration after exposure to PNP and possibly tease apart the effect of duration of exposure to the current light regime on the effects we describe here.

Unexpectedly, the effect of PNP on free-running period appeared rather small or even absent. In this context, we could not reproduce the previously found difference in free-running period caused by a long PNP (Ciarleglio et al., 2011), but we did observe an increase in variance in the same direction: a number of LP-developed animals did indeed show a short free-running rhythm, and we did see a larger variation in free-running period in this group compared to animals raised in SP PNP. This slight discrepancy between the 2 studies may be because in the previous study only the first 3 days in DD were analyzed (Ciarleglio et al., 2011), whereas here the period was determined later and over a longer time frame of 10 days. On the other hand, a different mouse strain may have been used in the Ciarleglio et al. (2011) study, indicating that effects of PNP on adult rest-activity patterns are present in different mouse strains, emphasizing the translational value of the similarities found between the 2 studies. With the data gathered, we have not been able to determine the origin of the variability in period in the long photoperiod males. There is no association between nest of origin and adult free-running behavior and the short-rhythm males come from different mothers, spread over different nests. It is remarkable that this effect is absent in females, as all of the females came from the same nest as the males. This suggests that female mice might be more resistant to the effect of PNP on free-running rhythms. We found a higher intradaily variability and a lower rhythmic strength in our C3H female mice compared to males. This is in contrast to what has been shown before in the C57BL/6 mouse strain where within-day activity in female mice is less varied compared to males (Levy et al., 2023; Smarr et al., 2017; Smarr and Kriegsfeld, 2022). With the current data, we conclude this difference in intradaily variability between the sexes is likely strain dependent.

When analyzing vigilance state distribution across the day, we found that LP-developed animals spent less time awake and more time in NREM sleep during the inactive (light) phase. Although this effect is not significant in the 1-h values, it is significant in the 12-h light period total. The difference between the groups in NREM sleep in the light period is approximately 7.2%, translating to approximately 1-h difference in the entire 12-h inactive phase. Previously, an effect of PNP on dorsal raphe nuclei neuronal activity and serotonin and noradrenalin content in midbrain areas was observed, with higher serotonin and noradrenalin levels in animals raised in a long photoperiod (Green et al., 2015). It is established that noradrenalin and serotonin play an important role in wake maintenance (reviewed in Monti, 2011; Saper et al., 2005), and it would therefore be expected that LP animals show an increase in waking, but we found the opposite. The full scope of influence of serotonin on sleep and circadian regulation is not known and therefore we cannot exclude the involvement of serotonin in other long-term effects of PNP and changes in sleep patterns. We conclude that the difference in sleep between the PNP-exposed animals is probably not caused by a direct effect of these monoamines on sleep.

We also did not observe any effects of PNP on the power density spectra, or on the response to sleep deprivation. The latter indicates that sleep homeostatic mechanisms (Borbély et al., 2016) are not significantly affected by PNP. This is consistent with the SWA analysis, where animals from both PNPs show only limited differences in relative SWA over time and no differences in the SWA response to sleep deprivation. Therefore, we consider it more likely that the PNP difference in amount of NREM sleep and waking originates from the circadian system.

Taken together, we see prolonged differences in behavioral locomotor activity and sleep in female and male mice in adulthood after exposure to different photoperiods when growing up. We conclude that PNP programs a developing mammal for future external conditions and with that changes the physiology of relevant nuclei, including the SCN, the nucleus accumbens, or the DRN, which results in permanent changes in sleep and the rest-activity cycle.

## Supplemental Material

sj-tif-1-jbr-10.1177_07487304241302547 – Supplemental material for Perinatal Photoperiod Has Long-Term Effects on the Rest-Activity Cycle and Sleep in Male and Female Mice Supplemental material, sj-tif-1-jbr-10.1177_07487304241302547 for Perinatal Photoperiod Has Long-Term Effects on the Rest-Activity Cycle and Sleep in Male and Female Mice by Rick van Dorp and Tom Deboer in Journal of Biological Rhythms
