# Supplementary figures and images for: Perinatal Photoperiod Has Long-Term Effects on the Rest-Activity Cycle and Sleep in Male and Female Mice
Source: J Biol Rhythms. 2024 Dec 18;40(1):62–75. doi: 10.1177/07487304241302547 (PMC11834332; doi:10.1177/07487304241302547)

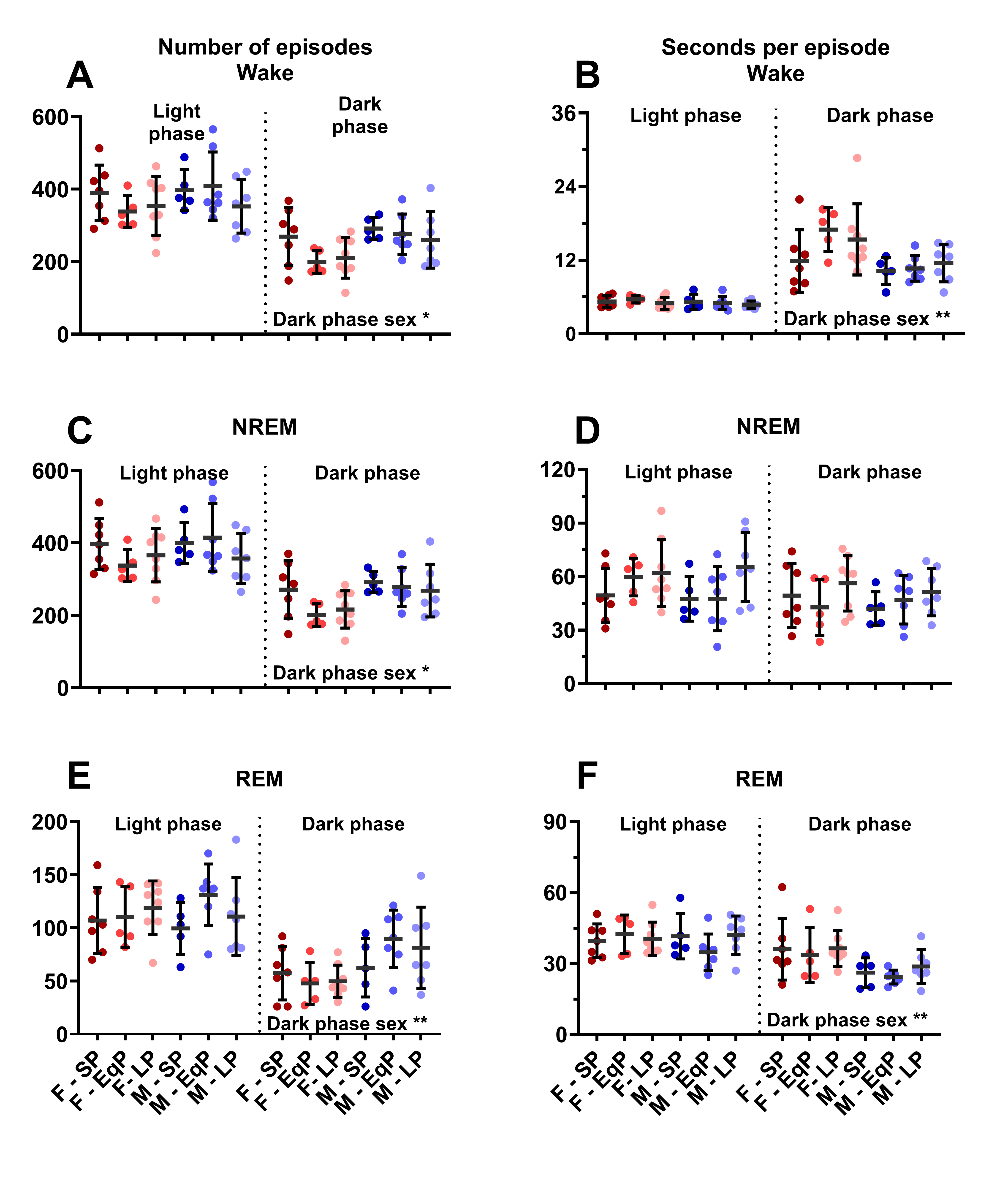

Supplement: sj-tif-1-jbr-10.1177_07487304241302547 – Supplemental material for Perinatal Photoperiod Has Long-Term Effects on the Rest-Activity Cycle and Sleep in Male and Female Mice [file sj-tif-1-jbr-10.1177_07487304241302547.tif]
